# Salivary Pellicle Modification with Grape-seed Extract: In Vitro Study on the Effect on Bacterial Adhesion and Biofilm Formation

**DOI:** 10.3290/j.ohpd.b1453013

**Published:** 2021-06-01

**Authors:** Thiago Saads Carvalho, Dea Muçolli, Sigrun Eick, Tommy Baumann

**Affiliations:** a Senior Research Associate and Senior Lecturer, Department of Restorative, Preventive and Pediatric Dentistry, School of Dental Medicine, University of Bern, Bern, Switzerland. Idea, conceptualisation, wrote the paper, critically reviewed the paper.; b Assistant Dentist, Department of Restorative, Preventive and Pediatric Dentistry / Department of Periodontology, School of Dental Medicine, University of Bern, Bern, Switzerland. Conceptualisation, performed the experiments, wrote the paper, critically reviewed the paper.; c Professor, Department of Periodontology, School of Dental Medicine, University of Bern, Bern, Switzerland. Idea, conceptualisation, performed the statistical analyses, contributed to the discussion, critically reviewed the paper.; d Research Associate, Department of Restorative, Preventive and Pediatric Dentistry, School of Dental Medicine, University of Bern, Bern, Switzerland. Idea, conceptualisation, contributed to the discussion, critically reviewed the paper.

**Keywords:** biofilms, caries, dental pellicle, enamel, fluoride, grape-seed extract, pellicle modification

## Abstract

**Purpose:**

Grape-seed extract (GSE) contains polyphenols that readily adhere to proteins and modify the acquired enamel pellicle (AEP). The first step in biofilm formation is bacterial adhesion to the AEP-covered enamel. The aim of this in vitro study was to test whether AEP modification with GSE, fluoride (F^-^), or their combination (GSE+F^-^) modulates bacterial adhesion, biofilm metabolism and composition, or cariogenic demineralisation of the enamel.

**Materials and Methods:**

The study comprised 3 parts: 1) single-strain *Streptococcus gordonii* species, 2) a five-species biofilm model, or 3) biofilm (re-)formation using the five-species biofilm model after removal of initial biofilm with toothbrushing. Human whole-mouth stimulated saliva was used to form an AEP on human enamel specimens. The AEP was incubated in water (control), or modified with GSE, F^-^, or GSE+F^-^. Bacterial adhesion, biofilm diversity, metabolic activity, biofilm mass, and cariogenic demineralisation (surface hardness) of enamel were assessed after incubation in bacterial broths after 4 h or 22 h. Differences between groups were analysed with one-way ANOVA and post-hoc Bonferroni tests.

**Results:**

GSE and GSE+F^-^ statistically significantly decreased single-strain *S. gordonii* adhesion, but had no relevant influence when the five-species biofilm model was used. In the biofilm (re-)formation model, GSE reduced bacterial adhesion compared to GSE+F^-^, while F^-^ caused less cariogenic demineralisation than was found in the control group.

**Conclusion:**

AEP modified with GSE retards *S. gordonii* adhesion, but it does not influence the formation, metabolism and composition of a cariogenic multi-species biofilm.

Caries is the most common disease of the dental hard tissue, burdening children and adults alike.^20^ The disease is defined as a local destruction of the minerals caused by cariogenic microorganisms that produce organic acids when supplied with fermentable carbohydrates. The microorganisms, however, rarely adhere directly onto the bare enamel devoid of saliva; rather, this adherence occurs on dental surfaces covered with the acquired enamel pellicle (AEP).^23^ The AEP is formed initially from potent interactions between proteins from the saliva and the tooth surface, forming a strongly-adhered bacteria-free protein layer. Further protein-protein interactions modulate the progression of AEP formation, which culminates in a mature pellicle composed mainly of statherin, amylase, proline-rich proteins (PRPs), mucins, among other proteins.^30^ Some of these proteins contain receptors that allow adhesion of bacteria,^13^ so that AEP is an integral part of oral homeostasis, directly influencing initial bacterial colonisation.^23^ Bacterial adherence begins with short-range physicochemical interactions between specific ligands on the bacterial cells and the receptors in the AEP.^13^

Considering this interplay, any modification of the AEP could have a direct impact on the adhesion of bacteria, which, in turn, could eventually modify the quality of the biofilm and lead to either an increase of biofilm (and ultimately more caries) or a decrease in biofilm (and ultimately a protective effect). Several substances have the ability to modify the AEP, including oils,^17^ proteins^4^ and plant extracts.^39^ The latter can do this largely because they contain polyphenols, which have the ability to form strong interactions with the salivary proteins,^6,9,33^ increasing the AEP thickness^19^ and improving its acid-resistance and protective qualities.^39^

Grape-seed extract (GSE) is a polyphenol-rich plant extract that has been gaining attention in dentistry. Grapes belong to the group of plants that contain high concentrations of tannins, so GSE is of great interest in caries preventive research. It seems to promote remineralisation,^5^ as well as being able to significantly inhibit single-strain bacteria such as *Streptococcus mutans*, or multi-species biofilms.^7^ However, its biofilm-inhibiting mechanism of action is not yet fully known, and the present study could help elucidate this question. We investigated whether the salivary pellicle modification with GSE, fluoride, or their combination will have an influence on: 1) bacterial adhesion of a single strain, 2) the formation (bacterial adhesion), composition, quantification and metabolic activity of a cariogenic multi-species biofilm, as well as the cariogenic demineralisation caused by the biofilm, 3) (re-)formation, composition, quantification and metabolic activity of a multi-species cariogenic biofilm and cariogenic demineralisation of enamel after mechanical removal (toothbrushing) of an initial biofilm.

## Materials and Methods

The present study used human teeth and saliva. It was carried out in accordance with the approved guidelines and regulations of the local ethics committee (Kantonale Ethikkommission). The teeth and saliva were pooled, and as the ethics committee categorises these pooled samples as “irreversibly anonymised”, no previous ethical approval was required.

### Preparation of Human Enamel Specimens

The human teeth used for this study were taken from a pooled biobank. They had been extracted by dental practitioners in Switzerland (no water fluoridation) and stored in 2% chloramine-T trihydrate solution.

The enamel specimens were prepared by embedding the teeth in acrylic resin, followed by grinding and polishing their surface (Struers Knuth-Rotor 2 and LaboPol 21; Ballerup, Denmark) until the outermost 200 µm of enamel was removed. This resulted in smooth, highly polished and planar parallel specimens. The enamel specimens were stored in a mineral solution (1.5 mmol/l CaCl_2_, 1.0 mmol/l KH_2_PO_4_, 50 mmol/l NaCl, pH 7.0) until the time of the experiment. Immediately prior to the experimental procedures, the initial surface microhardness (SMH) of all enamel specimens was measured. Potential contaminating bacteria on the specimens were killed by boiling, then incubation in brain-heart infusion broth (BHI; Biomérieux, Marcy l’etoile, France) at 37°C with 10% CO_2_ for 24 h, and then boiling a second time.

### Collection of Whole-mouth Stimulated Human Saliva

Whole-mouth stimulated human saliva was collected from healthy adult volunteers (n ≈ 30). They chewed on paraffin tablets for 10 min and collected their saliva in chilled vials. The saliva was immediately pooled, then centrifuged (20 min, 4°C, 4000 g). After collecting the supernatant, the fluid was exposed to UV radiation for 30 min to kill microorganisms, after which this saliva was divided into aliquots and stored at -80°C. The sterility of the saliva was tested by cultivation. Individual aliquots of saliva were defrosted immediately prior to the experiment and used.

Similar to the teeth used in the experiment, the pooled saliva is also categorised as ‘irreversibly anonymised’, and no previous ethical approval was necessary. Nevertheless, the saliva donors gave their informed spoken consent to use the saliva for research purposes.

### Salivary Pellicle Formation and Modification

After defrosting the sterile saliva, an aliquot of 50 µl was placed on each enamel specimen. AEP was allowed to form for 2 h (at 37°C), after which the enamel specimens were incubated for 30 min in one of four solutions for AEP modification, according to the following experimental groups.

1. Control group: non-modified pellicle layer (only deionised water was used);
2. Extract group (GSE): pellicle layer was modified with a 2 mg/ml grape-seed extract solution;
3. Fluoride group (F^-^): pellicle modified with a 500-ppm fluoride (from sodium fluoride) solution;
4. Extract and fluoride group (GSE+F^-^): pellicle modified with a solution containing 2 mg/ml grape-seed extract solution and 500 ppm fluoride.

All solutions (and deionised water) were freshly prepared under sterile conditions prior to the experimental procedures.

### Surface Microhardness Measurement

Surface microhardness (SMH) was measured with a Knoop diamond under a 50-g load and dwell time of 10 s (UHL VMHT Microhardness Tester, UHL Technischer Mikroskopie; Aßlar, Germany). Six indentations were made 50 μm apart, and the average of these six indentations was considered as the SMH value for the specimen. For statistical analyses, relative SMH (rSMH) was calculated using the formula: rSMH = (SMH_final_ / SMH_initial_) x 100.

### Bacterial Strains

The bacterial strains used in this study were: *Streptococcus gordonii* ATCC 10558, *Streptococcus mutans* ATCC 25175, *Streptococcus sobrinus* ATCC 12104, *Lactobacillus acidophilus* ATCC 11975 and *Actinomyces naeslundii* ATCC 12104. The strains were passaged for 24 h on tryptic soy agar (TSA) plates (Oxoid; Basingstoke, UK) with 5% sheep blood, 10% CO_2_, at 37°C.

### Bacterial Adhesion Assays and Cariogenic Demineralisation

Quantification of the biofilm was performed in accordance with previous studies.^22,27^ Since the biofilm is made up of bacterial cells (living and dead) and, to a large degree, extracellular matrix of exopolysaccharides (EPS), the analyses in the present experiment included: bacterial adhesion, biofilm mass (total mass including living and dead cells, as well as the EPS) and metabolic activity (mostly related to the living bacterial cells within the biofilm). The methods used here follow a previous study,^3^ and were carried out in 3 parts: 1) analysis of bacterial adhesion using a single bacterial species; 2) analysis of bacterial adhesion and cariogenic demineralisation using a five-species biofilm; 3) analysis of further bacterial adhesion and cariogenic demineralisation after mechanical removal of initial biofilm with a toothbrush.

#### 1. Analysis of bacterial adhesion using a single bacterial species

For this experimental setup, *S. gordonii* was used as the single bacterial species. A total of 48 enamel specimens were distributed into the four experimental groups (n=12), and the experiment was made in duplicate (twice with 6 specimens each). AEP formation and modification were carried out as described previously. The specimens were then contaminated with *S. gordonii* in a bacterial suspension (OD600= 0.1; equivalent to 108 bacteria/ml) in Dulbecco’s modified Eagle’s medium (DMEM; Gibco, Invitrogen) and incubated (37°C; 10% CO_2_; 30 min). In this experiment, we tested bacterial adhesion to the enamel specimens. This was quantified by determining bacterial counts (colony forming units [cfu]). For that, the specimens were washed once with 0.9% w/v NaCl solution to remove non-adhered bacteria, then the enamel surface was scraped with a cotton swab to remove the adhered biofilm, and the swabs were placed and dispersed in 1 ml 0.9% (w/v) NaCl solution. This suspension was then serially diluted 1:10 and plated onto tryptic soy agar with 5% sheep blood and incubated (10% CO_2_, 37°C, 72 h), after which the cfu were counted using an aCOLyte SuperCount colony counter (Synbiosis; Cambridge, UK).

#### 2. Analysis of bacterial adhesion and cariogenic demineralisation using a five-species biofilm

A total of 96 enamel specimens were used: 48 specimens (12 per group) were used to verify bacterial adhesion after 4 h of incubation; 48 specimens (12 per group) were used for biofilm formation and cariogenic demineralisation after 22 h of incubation. Again, the experiment was carried out in duplicate (with 6 specimens per group per experimental run).

The specimens were then submitted to AEP formation and modification, as previously described, and later contaminated with a five-species bacterial suspension consisting of a mixture of: *S. gordonii, S. mutans, S. sobrinus, L. acidophilus* and *A. naeslundii* (McFarland 4 in 0.9% w/v NaCl) in a ratio of 1:1:2:2:3, before adding a 1:9-ratio of brain-heart infusion (BHI) broth (BioMérieux; Marcy l’Etoile, France) with 5% sucrose and incubating for 4 h at 37°C with 10% CO_2_.

After the 4-h incubation period, the 48 specimens used for bacterial adhesion were removed from the experiment and treated for CFU counts. We then tested:

- Biofilm formation (bacterial adhesion): the adhered bacteria were removed with sterile cotton swabs soaked with NaCl (0.9%), and this solution was serially diluted 1:100 and plated onto tryptic soy agar, and incubated as before. The cfu counts were recorded.
- Biofilm composition: the cfu were counted considering the different bacterial strains.
- Quantification and metabolic activity: the suspension containing the biofilm was used to analyse the metabolic activity and the biofilm mass, as described in previous studies.^22,27^ Metabolic activity was assessed with a resazurin-based redox indicator, Alamar blue. For that, 5 µl of Alamar blue (alamarBlue reagent, Thermo Fisher Scientific; Waltham, MA, USA) was mixed with 100 µl of nutrient medium and added to the suspended biofilm. The mixture was incubated for 1 h at 37°C and analysed with an absorbance microplate reader (ELx808, Biotek Instruments; Winooski, VT, USA). Differences of absorbances at 600- to 570-nm wavelengths were calculated. For biofilm mass, the suspended biofilm was fixed (60°C; 1 h) and stained for 10 min with 50 µl of 0.06% (w/v) crystal violet (Sigma-Aldrich; St Louis, MO, USA) per well. The staining was then quantified using the absorbance microplate reader at a wavelength of 600 nm.

The other 48 specimens used for bacterial adhesion and cariogenic demineralisation were removed from the BHI broth with 5% sucrose medium and placed in BHI containing phosphate buffer (0.021 mol/l KH_2_PO_4_; 0.016 mol/l Na_2_HPO_4_). The specimens were left to incubate for a further 18 h (37°C; 10% CO_2_), for a total incubation time of 22 h. Afterwards, we checked biofilm formation, composition, and quantification and metabolic activity, as described above, but for bacterial count, the NaCl (0.9%) solution was serially diluted 1:1000.

These enamel specimens were also submitted to surface microhardness measurements to assess enamel demineralisation. SMH was measured before (SMH_initial_) and after exposure to bacteria (SMH_final_).

#### 3. Biofilm (re-)formation and analysis of further bacterial adhesion and cariogenic demineralisation after mechanical removal of initial biofilm with a toothbrush

For this assay, a total of 48 enamel specimens were distributed into these four experimental groups (12 specimens/group). Likewise, it was carried out in duplicate (with 6 specimens per group per experimental run). Initially, enamel surface microhardness (SMH_initial_) was measured, then enamel specimens were subjected to AEP formation and modification. Afterwards, they were contaminated with the five-species bacterial suspension, as described in part 2.

The specimens were incubated in the bacterial mixture for a total of 22 h (4 h in BHI with sucrose and 18 h in BHI with phosphate buffer), after which the biofilm was mechanically removed by toothbrushing. For that, the enamel specimens were individually brushed with soft toothbrushes (force: 1.5 ± 0.05 N; movement: 2 strokes/second for 10 s). Individual toothbrushes were used for the individual groups. After brushing, the specimens were dipped into 0.9% NaCl, and were once again submitted to AEP formation and modification (2 h, as described above), contamination with the five-species bacterial suspension, and incubation for another 22 h (4 h in BHI with sucrose and 18 h in BHI with buffer). Finally, assessment was performed as before: biofilm (re-)formation (bacterial adhesion), biofilm composition, and quantification and metabolic activity, as well as cariogenic demineralisation.

### Statistical Analysis

Results for each part of the study were analysed separately. One-way ANOVA with post-hoc Bonferroni tests were carried out for total cfu counts (log10), amount (%) of cariogenic streptococci (*S. mutans, S. sobrinus*) and *L. acidophilus*, metabolic activity of the biofilm, biofilm mass, and enamel surface microhardness.

## Results

### Analysis of Bacterial Adhesion using a Single Bacterial Species

**Fig 1:**
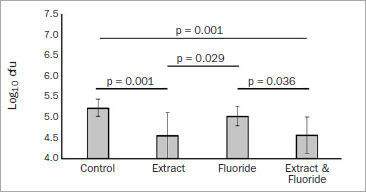
Assay of initial bacterial adhesion using a single-species model: means and SD (error bars) of colony forming units (log10 cfu) using a single bacterial species (*S. gordonii*) model after 30 min incubation. Statistical differences between groups are presented by lines with the respective p-value.

Using only one bacterial strain (*S. gordonii* ATCC 10558), statistically significant differences between the groups were observed (p < 0.001). The number of cfu in both groups containing the extract (GSE, GSE+F) was significantly lower than the control or F^-^ groups (Fig 1).

### Analysis of Bacterial Adhesion and Cariogenic Demineralisation using a Five-species Biofilm

**Fig 2:**
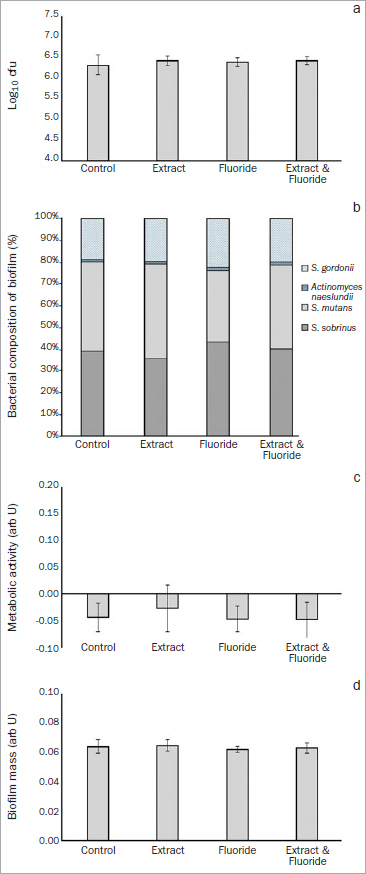
Assay of the formation, composition and metabolism of a cariogenic biofilm using a five-species biofilm model. Results after 4-h incubation in cariogenic medium: a. means and SD (error bars) of colony forming units (log10 cfu); b. proportion of different bacterial species in the biofilm (darker areas indicate cariogenic microorganisms; lighter [patterned] areas indicate non-cariogenic microorganisms); c. means and SD (error bars) of biofilm metabolic activity; d. means and SD (error bars) of biofilm mass. arb U: arbitrary units.

**Fig 3:**
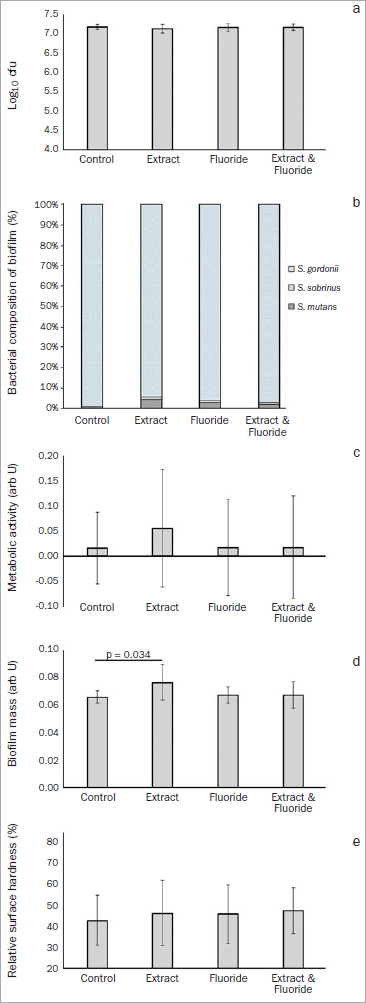
Assay of the formation, composition and metabolism of a cariogenic biofilm using a five-species biofilm model. Results after 22 h incubation (4 h in cariogenic medium, and 18 h in non-cariogenic medium): a. means and SD (error bars) of colony forming units (log10 cfu); b. proportion of different bacterial species in the biofilm (darker areas indicate cariogenic microorganisms; lighter [patterned] areas indicate non-cariogenic microorganisms); c. means and SD (error bars) of biofilm metabolic activity; d. means and SD (error bars) of biofilm mass; e. relative surface hardness. Statistical differences between groups are shown by lines with the respective p-value.

In the five-species biofilm (Figs 2 and 3) we analysed the following after 4 h and 22 h of incubation:

**Biofilm formation:** The amount of bacterial adhesion to the enamel increased by more than a 1 log10 in comparison to when one bacterial strain was used, with values around 5.0 log10 cfu for *S. gordonii* (Fig 1) to values over 6.0 or 7.0 log10 cfu for the five-species biofilm (Figs 2a and 3a, respectively). There were no statistically significant differences in bacterial adhesion between the groups, either after 4 h of incubation in a cariogenic medium (Fig 2a; p = 0.288) or after 22 h of incubation (Fig 3a; p = 0.607).

For the enamel specimens incubated for 4 h in a cariogenic medium, we observed:

- Biofilm composition (Fig 2): Almost 80% of the bacteria that adhered to the enamel surface were cariogenic (either *S. sobrinus* or *S. mutans*), and no significant differences were observed between the groups (p = 0.962).
- Quantification and metabolic activity: Statistically significant differences were found neither in metabolic activity of the biofilm (Fig 2c; p = 0.383) nor in biofilm mass (Fig 2d; p = 0.256).

For specimens incubated for 22 h, we observed:

- **Biofilm composition:** The cariogenic species portion of the bacteria adhered to enamel drastically decreased in comparison to the 4-h incubation period, with the vast majority of adhered bacteria being *S. gordonii,* but still no statistically significant differences were observed between the groups (p = 0.437).
- **Quantification and metabolic activity:** No statistically significant difference existed in the metabolic activity of the biofilm (Fig 3c; p = 0.702) between the groups, but the groups presented statistically significant differences in terms of biofilm mass (Fig 3d; p = 0.022). The GSE group presented statistically significantly greater biofilm mass than did the control group (p = 0.034). After 22 h of incubation, all groups presented enamel demineralisation to a similar degree (Fig 3e; p = 0.854), with relative enamel surface hardness decreasing from 100% to around 40%-50%.

### Analysis of Biofilm (Re-)formation and Cariogenic Demineralisation after Mechanical Removal of Initial Biofilm with Toothbrush

**Fig 4:**
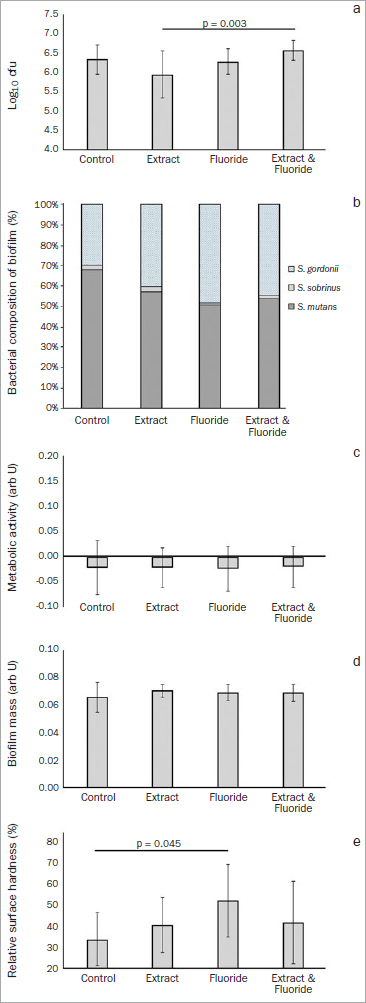
Assay of the (re-)formation of a cariogenic biofilm on enamel after mechanical removal of an initial biofilm: a. means and SD (error bars) of colony forming units (log10 cfu); b. proportion of different bacterial species in the biofilm (darker areas indicate cariogenic microorganisms; lighter [patterned] areas indicate non-cariogenic microorganisms); c. means and SD (error bars) of biofilm metabolic activity; d. means and SD (error bars) of biofilm mass; e. relative surface hardness. Statistical differences between groups are shown by lines with the respective p-value.

- **Biofilm formation:** After toothbrushing, (re-)formation of the biofilm differed between the groups (Fig 4a; p = 0.006), with lower cfu counts in the GSE group than in the GSE+F^-^ group (p = 0.003).
- **Biofilm composition:** The bacterial composition of the biofilm (re-)formed on the enamel specimens were largely made up of *S. gordonii,* but *S. mutans* was also present, and no statistically significant differences were observed between the groups (Fig 4b; p = 0.613).
- **Quantification and metabolic activity:** No differences were found in the metabolic activity of the biofilm (Fig 4c; p = 0.998) or in the biofilm mass (Fig 4d; p = 0.445). However, statistically significant differences were seen in the cariogenic demineralisation of the enamel: demineralisation was statistically significantly greater in the control group than in the F^-^ group (Fig 4e; p = 0.045).

## Discussion

Carious lesions are the detrimental outcome of the acid produced by oral biofilms. Thus, a major research focus in dentistry is on bettering the understanding of oral biofilms and means of maintaining their ecological balance. The first step in biofilm formation is the adhesion of the microorganisms to the pellicle-covered enamel. Initially, the adhesion is based on electrostatic attractions; at more advanced stages, chemical forces such as hydrogen bonds, hydrophobic interactions, calcium bridges, and van der Waals forces become more dominant.^13^ The process of adhesion begins when planktonic bacterial cells bind to specific proteins present in the AEP, among which are the proline-rich proteins (PRPs) and α-amylase.^13^ It is therefore speculated that any compound which binds to these proteins could modulate the process of bacterial adhesion.

Proanthocyanidins are polyphenols, which belong to the group of compounds that can readily bind to AEP proteins. They are found in a wide range of plants, with particularly high concentrations in flowers, leaves, nuts, fruits, and some seeds (such as grape seed).^31^ They have a complex structure formed from monomeric (flavan-3-ol) molecules bound together, arranged as dimers (2 monomeric units), oligomers (with a few monomeric units), or forming a polymeric structure (with up to 60 monomeric units). In proanthocyanidins from grapes, the most common flavan-3-ol monomeric units are (epi)catechin, (epi)afzelechin, and (epi)gallocatechin.^38^

Proanthocyanidins have a particularly high affinity towards α-amylase and PRPs. Their affinity is higher towards the latter, probably because they have numerous proline amino acids in their structure. There are two main mechanisms involved in the interaction between the polyphenolic compounds and proline:^1,28^ 1. proline contains a pyrrolidine ring, which cannot form hydrogen bonds with the oxygen atoms that form the peptide bonds in protein molecules; thus, the oxygen atoms in peptide bonds are “free” to readily form hydrogen bonds with the hydroxyl groups of polyphenols; 2. proline is a hydrophobic amino acid, able to form hydrophobic bonds with the hydrophobic ring structures of the polyphenols. PRPs also have a coiled structure that probably contains more proline amino acids on its outer surface which create more binding sites for the polyphenols. In contrast, α-amylase has less proline than do PRPs. It also has a globular conformation, which means it possesses an even lower number of proline amino acids on its outer surface, in turn leading to fewer binding sites for the polyphenolic molecules.^1^

Some proanthocyanidins, on the other hand, contain the galloyl moiety that grants the molecule a greater affinity towards α-amylase.^34^ This is the case in the oligomeric proanthocyanidins from grape-seed extracts, which are mostly made up of epicatechin gallate, a galloylated monomeric unit.^12^ It is therefore likely that the galloyl functional group in the oligomeric proanthocyanidins from the GSE could interact with both PRPs and α-amylase in the AEP, thus affecting bacterial adhesion.

Remarkably, our results show that bacterial adhesion was only affected when the *S. gordonii* single bacterial strain was used, decreasing its adhesion when the AEP was modified with GSE. A similar outcome was not observed in the multi-species biofilm. On the one hand, these results were unexpected because GSE can also inhibit multi-species biofilms.^7^ But on the other hand, our results might shed some light on the inhibiting mechanism of GSE. In the vast majority of studies with GSE on bacterial inhibition, GSE and the bacteria were in direct contact with each other, either by adding the GSE to the bacteria medium,^11,41^ or by applying a GSE rinse solution intraorally and later assessing salivary levels of streptococci.^29^ In contrast, our experiment applied the GSE solution to the AEP; the bacterial suspension was never in direct contact with the solution. Thus, the polyphenols did not act on the bacteria themselves, but rather on the proteins of the AEP. This leads us to speculate that, when the oligomeric proanthocyanidins from GSE are freely available in the bacteria medium or in the oral cavity, the polyphenolic molecules are able to interact directly with the bacterial cells and modulate their metabolism. They inhibit glucosyltransferase activity in *S. mutans,*^8,21^ which in turn causes fewer polysaccharides to be produced, and fewer polysaccharides promote less biofilm formation. However, when the polyphenols already interact with the AEP, such us in the present study, there might be an interaction with the early colonisers (as observed with the inhibition of *S. gordonii* adhesion), but not with the late colonisers, which are the most important for producing a biofilm matrix.

Furthermore, the anti-caries effect of GSE is best observed at a concentration of 2 mg/ml,^41^ which also yields the greatest anti-bacterial effect (minimum inhibitory concentration).^7,10,11,32^ Even though we used this concentration in our experiment, we observed no effect on the multi-species biofilm. Nevertheless, one can further assume a dilution of the GSE when it is adsorbed, and its final concentration in the APE will be lower. We also tested solutions containing 500 ppm fluoride, but observed no general protection from them, except in the assay of biofilm (re-)formation after toothbrushing, when the fluoride solution statistically significantly decreased enamel demineralisation. It is important to bear in mind that we applied the solutions only once, whereas the preventive effect of fluoride rather derives from its constant availability in the oral fluids. During the de- and remineralisation processes, fluoride is eventually incorporated into the enamel mineral crystal lattice.^36,37^ remineralising the tooth mineral. In fact, previous studies have also shown that GSE is also able to promote remineralisation, mostly in dentin.^2,5,11,26,35,40^

In the present study, the modification of the AEP was due to the strong interaction of polyphenolic compounds with proteins,^19^ which can ultimately lead to a thicker AEP.^18^ Such modified AEPs can have a protective effect against acid attacks.^16,39^ Hence, connecting all the above-mentioned concepts, we hypothesise that the mode of action of GSE in caries prevention could be threefold:

- Oligomeric proanthocyanidins adsorb onto the salivary pellicle and hinder the direct contact of acids with the tooth surface;^24^
- The oligomeric proanthocyanidin molecules already bound to the modified AEP have an effect on the initial adhesion of early colonisers such as *S. gordonii*, as observed in our results, but less effect on the adhesion of late colonisers. Nevertheless, the ‘freely available’ proanthocyanidin molecules in saliva (e.g. from using a mouthrinse containing GSE) can interact with the bacterial cells of late colonisers and inhibit glucosyltransferase, leading to fewer polysaccharides being produced, and consequently, less biofilm formation;
- The presence of oligomeric proanthocyanidins coupled with a regular availability of fluoride (e.g. from frequent oral hygiene measures) can intensify enamel remineralisation.

Despite the limitations of this in vitro study – which is far from representing the real in situ or in vivo situations – it still has its merits in that we used human teeth as the substrate and human saliva as the coating agent. The vast majority of studies testing the effect of plant extracts on bacterial adhesion either use glass beads or hydroxyapatite blocks as substrate, or artificial saliva/protein solutions as coating agents. Above all, these studies usually add the plant extracts directly to the bacterial strains.^25^ The use of either artificial saliva or protein solutions do not allow the formation of a clinically realistic AEP, likewise when glass beads or hydroxyapatite blocks are used; all of this influences bacterial adhesion. In this regard, our study probably led to a salivary pellicle that is more closely related to the clinical setting. It allowed us to test bacterial adhesion to a modified AEP without the direct influence of the extract on the bacteria. Some in situ studies have already shown that polyphenolic beverages lead to reduced reduced initial bacterial adhesion.^14,15^ In situ experiments are now necessary with the presented solutions to confirm the results in a clinical setting.

## Conclusion

A salivary pellicle modified with grape-seed extract is able to hinder the adhesion only of single-strain bacteria. However, the polyphenol molecules already bound to the pellicle do not influence the formation, metabolism or composition of a cariogenic multi-species biofilm.

## References

[ref1] Asano K, Shinagawa K, Hashimoto N (1982). Characterization of haze-forming proteins of beer and their roles in chill haze formation. ASBC Journal.

[ref2] Benjamin S, Sharma R, Thomas SS, Nainan MT (2012). Grape seed extract as a potential remineralizing agent: a comparative in vitro study. J Contemp Dent Pract.

[ref3] Carvalho TS, Halter JE, Mucolli D, Lussi A, Eick S, Baumann T (2020). Pellicle modification with casein and mucin does not promote in vitro bacterial biofilm formation. Oral Health Prev Dent.

[ref4] Cheaib Z, Lussi A (2011). Impact of acquired enamel pellicle modification on initial dental erosion. Caries Res.

[ref5] Cheng L, Li J, He L, Zhou X (2015). Natural products and caries prevention. Caries Res.

[ref6] Davies HS, Pudney PD, Georgiades P, Waigh TA, Hodson NW, Ridley CE (2014). Reorganisation of the salivary mucin network by dietary components: insights from green tea polyphenols. PLoS One.

[ref7] Delimont NM, Carlson BN (2020). Prevention of dental caries by grape seed extract supplementation: A systematic review. Nutr Health.

[ref8] Duarte S, Gregoire S, Singh AP, Vorsa N, Schaich K, Bowen WH (2006). Inhibitory effects of cranberry polyphenols on formation and acidogenicity of Streptococcus mutans biofilms. FEMS Microbiol Lett.

[ref9] Ferrer-Gallego R, Soares S, Mateus N, Rivas-Gonzalo J, Escribano-Bailon MT, de Freitas V (2015). New anthocyanin-human salivary protein complexes. Langmuir.

[ref10] Furiga A, Lonvaud-Funel A, Badet C (2009). In vitro study of antioxidant capacity and antibacterial activity on oral anaerobes of a grape seed extract. Food Chemistry.

[ref11] Furiga A, Roques C, Badet C (2014). Preventive effects of an original combination of grape seed polyphenols with amine fluoride on dental biofilm formation and oxidative damage by oral bacteria. J Appl Microbiol.

[ref12] Geny L, Saucier C, Bracco S, Daviaud F, Glories Y (2003). Composition and cellular localization of tannins in grape seeds during maturation. J Agric Food Chem.

[ref13] Hannig C, Hannig M (2009). The oral cavity –a key system to understand substratum-dependent bioadhesion on solid surfaces in man. Clin Oral Investig.

[ref14] Hannig C, Sorg J, Spitzmuller B, Hannig M, Al-Ahmad A (2009). Polyphenolic beverages reduce initial bacterial adherence to enamel in situ. J Dent.

[ref15] Hannig C, Spitzmuller B, Al-Ahmad A, Hannig M (2008). Effects of Cistus-tea on bacterial colonization and enzyme activities of the in situ pellicle. J Dent.

[ref16] Hertel S, Potschke S, Basche S, Delius J, Hoth-Hannig W, Hannig M (2017). Effect of tannic acid on the protective properties of the in situ formed pellicle. Caries Res.

[ref17] Ionta FQ, Alencar CRB, Val PP, Boteon AP, Jordao MC, Honorio HM (2017). Effect of vegetable oils applied over acquired enamel pellicle on initial erosion. J Appl Oral Sci.

[ref18] Joiner A, Muller D, Elofsson UM, Arnebrant T (2004). Ellipsometry analysis of the in vitro adsorption of tea polyphenols onto salivary pellicles. Eur J Oral Sci.

[ref19] Joiner A, Muller D, Elofsson UM, Malmsten M, Arnebrant T (2003). Adsorption from black tea and red wine onto in vitro salivary pellicles studied by ellipsometry. Eur J Oral Sci.

[ref20] Kassebaum NJ, Bernabe E, Dahiya M, Bhandari B, Murray CJ, Marcenes W (2015). Global burden of untreated caries: a systematic review and metaregression. J Dent Res.

[ref21] Koo H, Duarte S, Murata RM, Scott-Anne K, Gregoire S, Watson GE (2010). Influence of cranberry proanthocyanidins on formation of biofilms by Streptococcus mutans on saliva-coated apatitic surface and on dental caries development in vivo. Caries Res.

[ref22] Kwasny SM, Opperman TJ (2010). Static biofilm cultures of Gram-positive pathogens grown in a microtiter format used for anti-biofilm drug discovery. Curr Protoc Pharmacol.

[ref23] Marsh PD (2004). Dental plaque as a microbial biofilm. Caries Res.

[ref24] Niemeyer SH, Baumann T, Lussi A, Meyer-Lueckel H, Scaramucci T, Carvalho TS (2020). Salivary pellicle modification with polyphenol-rich teas and natural extracts to improve protection against dental erosion. J Dent.

[ref25] Palombo EA (2011). Traditional medicinal plant extracts and natural products with activity against oral bacteria: potential application in the prevention and treatment of oral diseases. Evid Based Complement Alternat Med.

[ref26] Pavan S, Xie Q, Hara AT, Bedran-Russo AK (2011). Biomimetic approach for root caries prevention using a proanthocyanidin-rich agent. Caries Res.

[ref27] Pirracchio L, Joos A, Luder N, Sculean A, Eick S (2018). Activity of taurolidine gels on ex vivo periodontal biofilm. Clin Oral Investig.

[ref28] Siebert KJ (1999). Effects of protein-polyphenol interactions on beverage haze, stabilization and analysis. J Agric Food Chem.

[ref29] Singla S, Malhotra R, Nd S, Saxena S (2018). Antibacterial efficacy of mouthwash prepared from pomegranate, grape seed and guava extracts against oral streptococci: an in vivo study. J Clin Pediatr Dent.

[ref30] Siqueira WL, Custodio W, McDonald EE (2012). New insights into the composition and functions of the acquired enamel pellicle. J Dent Res.

[ref31] Smeriglio A, Barreca D, Bellocco E, Trombetta D (2017). Proanthocyanidins and hydrolysable tannins: occurrence, dietary intake and pharmacological effects. Br J Pharmacol.

[ref32] Smullen J, Koutsou GA, Foster HA, Zumbe A, Storey DM (2007). The antibacterial activity of plant extracts containing polyphenols against Streptococcus mutans. Caries Res.

[ref33] Soares S, Vitorino R, Osorio H, Fernandes A, Venancio A, Mateus N (2011). Reactivity of human salivary proteins families toward food polyphenols. J Agric Food Chem.

[ref34] Sun LJ, Warren FJ, Gidley MJ (2019). Natural products for glycaemic control: polyphenols as inhibitors of alpha-amylase. Trends Food Sci Technol.

[ref35] Tang CF, Fang M, Liu RR, Dou Q, Chai ZG, Xiao YH (2013). The role of grape seed extract in the remineralization of demineralized dentine: micromorphological and physical analyses. Arch Oral Biol.

[ref36] ten Cate JM (2013). Contemporary perspective on the use of fluoride products in caries prevention. Br Dent J.

[ref37] ten Cate JM, Buzalaf MAR (2019). Fluoride mode of action: once there was an observant dentist. J Dent Res.

[ref38] Unusan N (2020). Proanthocyanidins in grape seeds: An updated review of their health benefits and potential uses in the food industry. J Funct Foods.

[ref39] Weber MT, Hannig M, Potschke S, Hohne F, Hannig C (2015). Application of plant extracts for the prevention of dental erosion: an in situ/in vitro study. Caries Res.

[ref40] Xie Q, Bedran-Russo AK, Wu CD (2008). In vitro remineralization effects of grape seed extract on artificial root caries. J Dent.

[ref41] Zhao W, Xie Q, Bedran-Russo AK, Pan S, Ling J, Wu CD (2014). The preventive effect of grape seed extract on artificial enamel caries progression in a microbial biofilm-induced caries model. J Dent.

